# New specific primers for amplification of the Internal Transcribed Spacer region in Clitellata (Annelida)

**DOI:** 10.1002/ece3.3212

**Published:** 2017-10-31

**Authors:** Yingkui Liu, Christer Erséus

**Affiliations:** ^1^ Department of Biological and Environmental Sciences University of Gothenburg Göteborg Sweden

**Keywords:** Hirudinida, Internal Transcribed Spacer region, Oligochaeta, polymerase chain reactions, primers

## Abstract

Nuclear molecular evidence, for example, the rapidly evolving Internal Transcribed Spacer region (ITS), integrated with maternally inherited (mitochondrial) COI barcodes, has provided new insights into the diversity of clitellate annelids. PCR amplification and sequencing of ITS, however, are often hampered by poor specificity of primers used. Therefore, new clitellate‐specific primers for amplifying the whole ITS region (ITS: 29F/1084R) and a part of it (ITS2: 606F/1082R) were developed on the basis of a collection of previously published ITS sequences with flanking rDNA coding regions. The specificity of these and other ITS primers used for clitellates were then tested in silico by evaluating their mismatches with all assembled and annotated sequences (STD, version r127) from EMBL, and the new primers were also tested in vitro for a taxonomically broad sample of clitellate species (71 specimens representing 11 families). The in silico analyses showed that the newly designed primers have a better performance than the universal ones when amplifying clitellate ITS sequences. In vitro PCR and sequencing using the new primers were successful, in particular, for the 606F/1082R pair, which worked well for 65 of the 71 specimens. Thus, using this pair for amplifying the ITS2 will facilitate further molecular systematic investigation of various clitellates. The other pair (29F/1084R), will be a useful complement to existing ITS primers, when amplifying ITS as a whole.

## 1 INTRODUCTION

**Figure 1:**
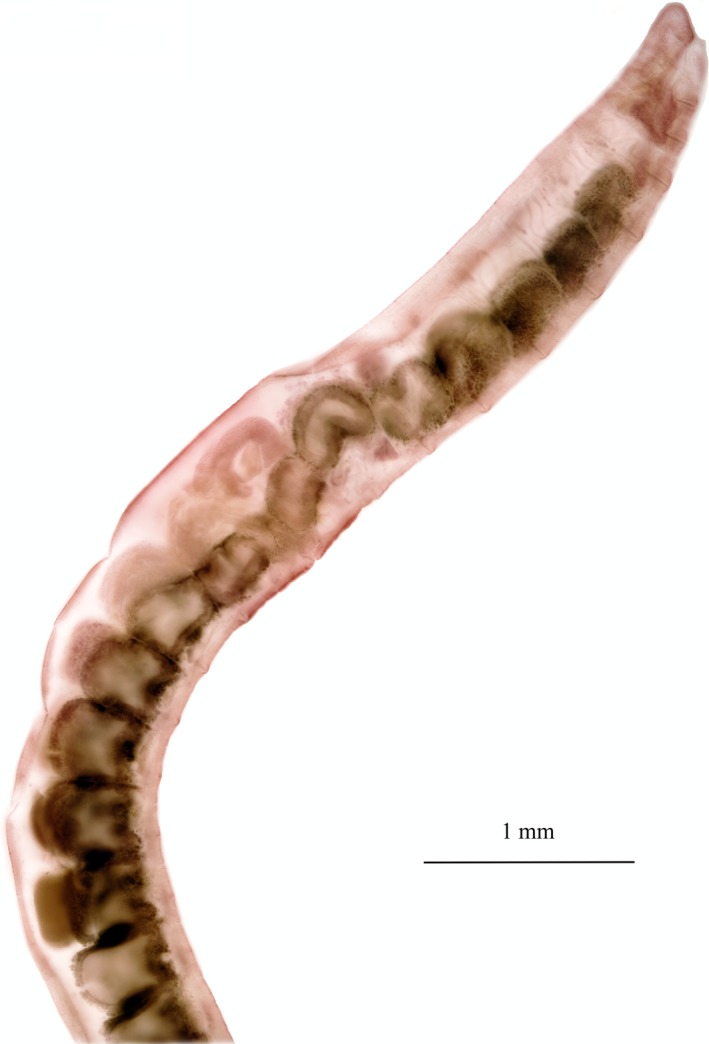
The head end of a typical freshwater member of Naididae (Clitellata), *Limnodrilus hoffmeisteri* Claparéde, 1862, today known to be a complex of cryptic species. The specimen is preserved and mounted on a microscope slide. The region of the clitellum (i.e., the “girdle”) is the slight widening of the body in about the middle of the picture

In molecular systematics, multilocus sequence data, both from mitochondrial and nuclear genomes, provide a better understanding of speciation than any single‐locus data (typically maternally inherited mitochondrial ones) (Dupuis, Roe, & Sperling, 2012; Mallo & Posada, 2016). As the analysis of a single‐locus data produces a gene tree rather than a species tree, such data should be integrated with nuclear evidence to establish species boundaries more accurately (Dasmahapatra, Elias, Hill, Hoffman, & Mallet, 2010; Kodandaramaiah, Simonsen, Bromilow, Wahlberg, & Sperling, 2013). This has been performed for many species of Clitellata (see Figure 1; they are segmented hermaphroditic annelid worms, bearing a unique clitellum (“girdle”) during sexual maturity, and many of them (earthworms, sludge worms, leeches) are important in agriculture, industry, environmental monitoring, and medicine (Elissen, Hendrickx, Temmink, & Buisman, 2006; Martin, Martinez‐Ansemil, Pinder, Timm, & Wetzel, 2008; Rodriguez & Reynoldson, 2011; Sket & Trontelj, 2008). Closely related clitellates are often difficult to distinguish morphologically, but molecular studies have shown that several well‐known morphotaxa, even those used as model organisms, are complexes of cryptic species (Erséus & Gustafsson, 2009; James et al., 2010; Römbke et al., 2016; Siddall, Trontelj, Utevsky, Nkamany, & Macdonald, 2007).

Using mitochondrial COI barcodes suggested for animals (Hebert, Ratnasingham, & de Waard, 2003) to validate and identify the currently >5,000 described species of Clitellata (Erséus, 2005), however, is still far from satisfactory (Trebitz, Hoffman, Grant, Billehus, & Pilgrim, 2015; Vivien, Wyler, Lafont, & Pawlowski, 2015). Such single‐locus data only reflect the history of one gene; however, they may still give hints of cryptic speciation by showing “barcoding gaps.” Therefore, in more comprehensive studies of species delimitation, COI data have been used to produce primary species hypotheses only, and the final species hypotheses have then been formulated based on congruence with hypotheses derived from independent nuclear markers (Kvist, Sarkar, & Erséus, 2010; Liu, Fend, Martinsson, & Erséus, 2017a; Martinsson & Erséus, 2014; Martinsson, Rhodén, & Erséus, 2017; Vivien et al., 2015).

One of these nuclear markers, the Internal Transcribed Spacer (ITS) region, has been commonly used in combination with COI in taxonomic works (Bucklin, Steinke, & Blanco‐Bercial, 2011; Coissac, Hollingsworth, Lavergne, & Taberlet, 2016; Raupach et al., 2010), as well as in studies of phylogeny, biogeography, and population genetics (De Wit & Erséus, 2010; Hallett, Atkinson, & Bartholomew, 2005; Trontelj & Sket, 2000; Trontelj & Utevsky, 2012; Villalobos et al., 2014). This region, which comprises two fast‐evolving spacers (ITS1 and ITS2) flanking the conserved 5.8S rDNA, has indeed been suggested as a universal DNA barcode marker for Fungi (Schoch et al., 2012), and a supplementary barcode for plants (Li et al., 2015; Pecnikar & Buzan, 2014). However, there has been a long debate about the relative value of ITS1 and ITS2 (Bazzicalupo, Balint, & Schmitt, 2013; Blaalid et al., 2013; Wang et al., 2015; Yao et al., 2010).

The ITS1 spacer seems to be more variable than ITS2, due to the frequent occurrence of indels (Edger et al., 2014; Martin & Rygiewicz, 2005; Nilsson, Kristiansson, Ryberg, Hallenberg, & Larsson, 2008; Rampersad, 2014). ITS1 is used in molecular identification of fungi in the publicly available databases UNITE (Koljalg et al., 2013) and ITSoneDB (Fosso et al., 2012), but the annotation and analyses of ITS1 of other taxonomic groups may be challenging. Because annotation is commonly performed by directly comparing new amplicons with those published sequences, however, the coverage of both the ITS1 and ITS2 regions in GenBank is often incomplete or incorrectly annotated. On the other hand, a comprehensive ITS2 database (Schultz et al., 2006) has facilitated the annotation of ITS2 sequences across many groups of organisms, by predicting their 5.8S‐28S interactions in a homology‐based structure modeling approach (Selig, Wolf, Müller, Dandekar, & Schultz, 2008). In particular, throughout the eukaryotes, the four helices in the secondary structure of ITS2 are consistent (Coleman, 2007; Gottschling & Plötner, 2004; Hausner & Wang, 2005; Schultz, Maisel, Gerlach, Muller, & Wolf, 2005), which is essential for successful excision of ITS2 from the precursor rDNA (Henras, Plisson‐Chastang, O'Donohue, Chakraborty, & Gleizes, 2015; Mullineux & Lafontaine, 2012). The rather conservative secondary structure of ITS2 makes it realistically suitable also for higher level systematics (Caisova, Marin, & Melkonian, 2011; Coleman, 2003; Marinho et al., 2012; Porras‐Alfaro, Liu, Kuske, & Xie, 2014; Salvi & Mariottini, 2017; Schultz et al., 2006). Knowledge of ITS2 secondary structure can improve the quality of an alignment using other carefully annotated sequences as a backbone (Katoh & Standley, 2013; Keller et al., 2010), which makes it possible to identify the consensuses motifs universally shared by closely related species (Pepato & Klimov, 2015). Thus, ITS2 may also provide sufficient information for cryptic species and young radiations (Bertrand et al., 2014; Coleman, 2009; Martinsson et al., 2017; Ruhl, Wolf, & Jenkins, 2010; Schill, Forster, Dandekar, & Wolf, 2010; Wiemmers, Keller, & Wolf, 2009), and estimation of gene flow within panmictic populations of deeply divergent mitochondrial lineages (Martinsson et al., 2017). Yao et al. (2010) even suggested that ITS2 should be used as a complementary locus for the identification of animals along with COI barcodes. Considering the general annotation and structure prediction tools provided by the ITS2 database (Schultz et al., 2006), it seems that ITS2, at present, is a more suitable nuclear marker than ITS1 for nonfungal groups such as clitellates.

**Figure 2:**
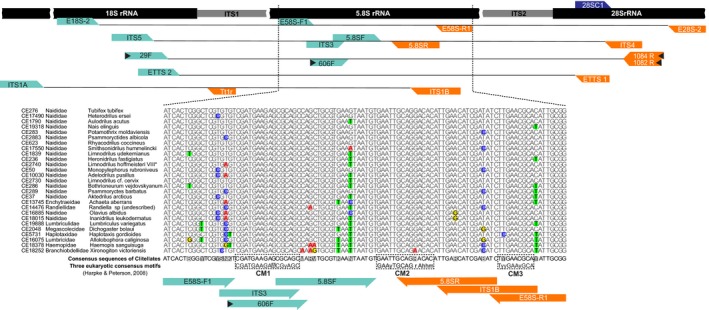
Diagram mapping primers for amplification of ITS2, and the ITS region as a whole, in clitellate worms. Forward (cyan arrows) and reverse primers (orange arrows) of newly designed (arrows with a black arrowhead inside) and previously published primers (without arrowhead) were marked. In addition, the commonly used primer 28SC1 (Jamieson et al., 2002; purple arrow) for amplifying 28S, the reverse of ETTS1, is also shown here. The alignment shows partial sequences of the 5.8S rDNA (located between the two Internal Transcribed Spacers, ITS1 and ITS2) of the 27 haplotypes found in our newly amplified complete ITS sequences, ranked by numbers of mismatches (high‐lighted). The location of three conservative motifs (CM1‐3), recognized for eukaryotes by (Harpke & Peterson, 2008), are also shown. *VIII refers to a cryptic species in the *L. hoffmeisteri* complex (Liu, Fend, et al. 2017a)

Various universal primer pairs (Figure 2 and Table 1) have been used for amplification of the entire or parts of the ITS region in clitellate studies. However, universal primers sometimes have low success rate in the polymerase chain reactions (PCR) (Oceguera‐Figueroa, 2012; Shekhovtsov, Golovanova, & Peltek, 2013; Trontelj & Utevsky, 2012; Vivien et al., 2015), due to poor specificity of these primers (Bellemain et al., 2010; Sipos et al., 2007). Furthermore, mismatches between primer and DNA templates might also introduce biases in PCR‐based high‐throughput Next Generation Sequencing (Aird et al., 2011; Deakin et al., 2014; Schirmer et al., 2015). Universal primers thus often have to be modified to make them suitable for amplifications of specific organisms (Bellemain et al., 2010; Cheng et al., 2016; Kohout et al., 2014; Toju, Tanabe, Yamamoto, & Sato, 2012). For example, Källersjö, Von Proschwitz, Lundberg, Eldenäs, and Erséus (2005) amplified ITS sequences of freshwater bivalves using the more bivalve‐specific forward primer MITS1F together with the universal primer ITS4, instead of using the primer pair ITS5/ITS4 (White, Bruns, Lee, & Taylor, 1990), which were originally developed for Fungi but are now used as a universal primer (see https://unite.ut.ee/primers.php). PCR failure may also be caused by intra‐individual polymorphism (Kook et al., 2015), which has been found, for example, in the European earthworm *Aporrectodea longa* (Martinsson et al., 2017).

**Table 1:** The list of published primers used for amplifying ITS sequences of clitellates

Primers pairs	Amplicons	Additional sequencing primers	References
ITS3/ITS4	ITS2	n/a	(Trontelj & Utevsky, 2005)
ITS5/ITS4	ITS	5.8SF/5.8SR	(Källersjö et al., 2005; Oceguera‐Figueroa, 2012)
E18S‐2/E28S‐2	ITS	E58S‐F1/E58S‐R1	(Shekhovtsov et al., 2013)
ITS1A/ITS1B	ITS1	n/a	(Kerans et al., 2004; Williams et al., 2013)
ETTS1/ETTS2	ITS	n/a	(Siqueira et al., 2013)
ITS3/ITS4 ITSbyk/ITS4 ITSkra/ITS4	ITS2	n/a	(Trontelj & Sket, 2000)
ITS1A/Tt1r	ITS1	n/a	(Hallett et al., 2005)

As yet, no clitellate‐specific ITS primers have been formally proposed. In this paper, two new pairs of primers specifically designed to amplify the whole ITS region and ITS2 spacer in clitellates are proposed. One of them (606F/1082R for ITS2) was successfully tested also by Martinsson et al. (2017), and Liu et al. 2017b.

## 2 MATERIAL AND METHODS

### 2.1 Primer design

In contrast to the fast‐evolving ITS1 and ITS2 spacers, the flanking 18S and 28S rDNA, as well as 5.8S rDNA between the two spacers, are more conserved and thus suitable as annealing regions for primers. An alignment was generated from a collection of 742 ITS sequences referred to Clitellata, that is, all those publicly available in GenBank (NCBI), and which include at least a part of 5.8S rDNA; several of them also include parts of 18S and/or 28S rDNA. Annotation and separation of ITS1, ITS2 and 5.8S rDNA are crucial for proper alignment, but aligning ITS sequences from divergent taxa may be problematic due to length variations (Alvarez & Wendel, 2003; Simmons & Freudenstein, 2003). Therefore, the three partitions of each downloaded ITS sequence were first identified using ITSx (Bengtsson‐Palme et al., 2013). In addition, boundaries of rDNAs were tested against the Rfam databases (Nawrocki et al., 2015), and the annotations of ITS2 were also checked using the Hidden Markov model (HMM) in the ITS2 database (*E*‐value < .001, metazoan) (Keller et al., 2009). Alignments of each ITS partition were conducted using the MAFFT V 7.017 plugin with default settings as implemented in Geneious 6.1.8. Based on the consensus sequence of this alignment, primer candidates were identified within the retained series of multiple conservative sites (each >14 nucleotides long), and two primer pairs with the highest possible scores, for ITS as a whole and ITS2, respectively, were identified using the software Oligo 7 (Rychlik, 2007). Heterozygosity within PCR primer binding sites do have negative effects for amplification, but in most cases, heterozygosity is more commonly found in ITS spacer sequences than in the short flanking rDNA sequences (see Martinsson et al., 2017).

### 2.2 Experimental verification of new primers

The universality of the new primers among clitellates was tested by PCR, amplifying specific fragments from 71 genomic DNA samples (47 genera, 11 families; Table 2); for extraction protocols, see Liu, Fend, et al.2017a. The samples were chosen to represent as many available families as possible, but also to cover several genera in the highly diverse family Naididae and to include some samples of very closely related species; three nominal naidids (*Doliodrilus tener, Limnodrilus grandisetosus*, and *L. rubripenis*) were even each represented by two specimens that are likely to be different (cryptic) species. A typical naidid, *Limnodrilus hoffmeisteri*, is shown in Figure 1. This mixture was chosen to obtain general information about ITS variability within both higher and lower taxa, which will facilitate a better annotation of new clitellate amplicons (as future reference sequences, for example, in secondary structure‐based analyses of ITS). In addition, samples that did not successfully amplify with the new primers were also tested using the universal primer pair ITS5/ITS4 without additional primers (see Table 1).

**Table 2:** Taxonomic sampling, collection sites and GenBank accession numbers of specimens used in this study. DNA sequences were derived from tissue samples from the posterior part of the worms

Specimen ID	Family name	Species	606F/1082R	29F/1084R	ITS1 (bp)	5.8S (bp)	ITS2 (bp)	GenBank	Voucher ID	Location and habits	Latitude	Longitude	Date	Collector
CE18252	Branchiobdellidae	*Xironogiton victoriensis* Gelder & Hall, 1990	**+**	**+**	>1,097	153	>810	KY982581	No voucher	Luxembourg, near Welscheid, Wark Brook, from a crayfish (*Pacificastaus leniusculus*)	49.880 N	6.044 E	15‐May‐2013	David Templeman
CE14346	Capilloventridae	*Capilloventer australis* Erséus, 1993	**+**	**−**	**−**	>92	747	KY982554	No voucher	Australia, Victoria, Acheron River (NE of Melbourne), gravel and sand	37.3526 S	145.7066 E	12‐April‐2012	C. Erséus & Richard Marchant
CE13745	Enchytraeidae	*Achaeta aberrans* Nielsen & Christensen 1961	**+**	**+**	>439	153	330	KY982545	SMNH 162129	Sweden, Västergötland, Vårgårda, Bergstena, near Lundagården Spring	58.069 N	12.689 E	28‐November‐2011	Christer Erséus, N. Bekkouche & Marcus Svensson
CE11317	Enchytraeidae	*Chamaedrilus sphagnetorum* (Vejdovský, 1878) (s.str.)	**+**	**−**	**−**	>70	248	KY982555 KF672519	SMNH 133623	Sweden, Närke, Hallsberg, Östansjö, Ögonakällan Spring	59.0389 N	15.0186 E	7‐April‐2011	Ainara Achurra & Christer Erséus
CE19554	Enchytraeidae	*Fridericia magna* Friend, 1899	**+**	**+**	>436	153	287	KY982559	ZMBN 110195	Norway, Möre og Romsdal, Tingvoll, Kanestraum, at ferry terminal (ferry across Halsfjorden)	63.0531 N	8.1233 E	13‐August‐2013	Christer Erséus
CE19299	Enchytraeidae	*Lumbricillus lineatus* (Müller, 1774)	**+**	**+**	>464	153	289	KY982569	ZMBN 107874	Norway, Sogn og Fjordane, Luster, Nes, seashore	61.3864 N	7.3691 E	12‐August‐2013	Christer Erséus
CE5731	Haplotaxidae	*Haplotaxis gordioides* (Hartmann, 1821)	**+**	**+**	>472	153	302	KY982561	SMNH 162130	Sweden, Västergötland, Göteborg, Vitsippsdalen (at Botanical Garden), wet soil	57.6752 N	11.9644 E	8‐April‐2009	Christer Erséus
CE18378	Hirudinidae	*Haemopis sanguisuga* (Linnaeus, 1758)	**+**	**+**	>322	153	381	KY982560	No voucher	Sweden, Västergötland, Vårgårda, Lången Lake, shallow water	58.011 N	12.582 E	27‐July‐2013	Christer Erséus
CE12000	Lumbricidae	*Allolobophora caliginosa* (Savigny, 1826)	**+**	**+**	>503	153	416	KY982547	ZMBN 108456	Norway, Telemark, Porsgrunn, Eidanger, Langansvegen	59.1162 N	9.7216 E	16‐June‐2011	Christer Erséus
CE16075	Lumbricidae	*Aporrectodea caliginosa* (Savigny, 1826)	**+**	**+**	>389	153	416	KY982549	ZMBN 108577	Norway, Nordland, Fauske, E of Törresvik, at Rd 80	67.2656 N	15.2939 E	17‐August‐2012	Endre Willassen & Christer Erséus
CE10969	Lumbriculidae	*Dorydrilus michaelseni* Piguet, 1913	**−**	**−**	**−**	**−**	**−**	**−**	SMNH 162131	England, Devon, Ivybridge, Higher Ludbrook Farm, spring	50.37 N	3.89 W	18‐March‐2010	Tim Jones
CE14379	Lumbriculidae	*Kincaidiana hexatheca* Altman, 1936	**+**	**+**	>546	153	313	KY982565	No voucher	USA, Oregon, Rock Creek (Portland)	45.5 N	122.9 E	27‐March‐2012	Sam James
CE19888	Lumbriculidae	*Lumbriculus variegatus* (Müller, 1774)	**+**	**+**	>638	153	312	KY982570	No voucher	Norway, Oslo, Majorstua, Vigelandsparken, stream near swimming pools	59.9281 N	10.7059 E	10‐October‐2012	Christer Erséus, Svante Martinsson & Yingkui Liu
CE17795	Lumbriculidae	*Stylodrilus heringianus* Claparède, 1862	**+**	**−**	**−**	>107	321	KY982578	No voucher	Sweden, Södermanland, Vingåker, Läppe, Hjälmaren Lake, sand and gravel	59.13 N	15.81 E	27‐July‐2012	Christer Erséus
CE2048	Megascolecidae	*Dichogaster bolaui* (Michaelsen, 1891)	**+**	**+/−**	>93	153	338	KY982556	SMNH 162132	Sweden, Västergötland, Göteborg, Tynnered, bathroom (apartment building)	57.64 N	11.89 E	27‐September‐2006	Daniel Gustafsson
CE713_1	Naididae	*Branchiura sowerbyi* Beddard, 1892	**−**	**−**	**−**	**−**	**−**	**−**	SMNH 160320	Sweden, Västmanland, Västerås, Mälaren Lake, Västeråsfjärden, Djuphamnen,	59.589 N	16.527 E	17‐September‐2003	Tommy Odelström
CE10030	Naididae	*Adelodrilus pusillus* Erséus, 1978	**+**	**+**	>490	153	385	KY982546	SMNH 162133	Sweden, Bohuslän, Strömstad, Brattebergsund (strait between Öddö and Tjärnö Islands), 8 m	58.894 N	011.163 E	14‐September‐2010	Christer Erséus
CE37	Naididae	*Aktedrilus arcticus* (Erséus, 1978)	**+**	**+**	>459	153	258	KY637025	No voucher	Sweden, Bohuslän, Strömstad, Tjärnö, beach in front of Research Station, intertidal sand	58.8755 N	11.1458 E	1‐August‐1997	Christer Erséus
CE1790	Naididae	*Aulodrilus acutus* Ohtaka & Usman, 1997	**+**	**+**	>426	153	377	KY637027	SMNH 160319	Cambodia, Kampong Chnang, Lake Tonle Sap	12.261 N	104.681 E	21‐May‐2005	Akifumi Ohtaka
CE14362	Naididae	*Aulodrilus japonicus* Yamaguchi, 1953	**+**	**+**	>515	153	389	KY982550	No voucher	Australia, Victoria, Acheron River (NE of Melbourne), gravel and sand	37.3526 S	145.7066 E	12‐April‐2012	C. Erséus & Richard Marchant
CE281	Naididae	*Aulodrilus pluriseta* Piguet, 1906	**+**	**−**	**−**	>64	288	KY637028	No voucher	Estonia, Rannu, Vörtsjärv Limnological Station, lab culture kept by Tarmo Timm	58.212 N	26.110 E	1‐December‐2000	Timm Tarmo
CE196_2	Naididae	*Baltidrilus costatus* (Claparède, 1863)	**+**	**+**	>715	153	481	KY637029	No voucher	Sweden, Bohuslän, Strömstad, Koster archipelago, subtidal sand,	58.875 N	11.080 E	1‐September‐2000	Christer Erséus
CE17439	Naididae	*Bathydrilus formosus* Erséus, 1986	**+**	**+**	>591	153	357	KY982551	SMNH 162134	Bahamas, Exuma, cut between Darby Island and Little Darby Island, 6 m, coarse sand	23.8559 N	76.2248 W	1‐April‐2013	Christer Erséus
CE17759	Naididae	*Bothrioneurum vejdovskyanum* Štolc, 1886	**+**	**+**	351	153	251	KY982552	SMNH 162135	Sweden, Södermanland, Vingåker, Läppe, Hjälmaren Lake, sand and gravel	59.13 N	15.81 E	27‐July‐2012	Christer Erséus
CE2213	Naididae	*Branchiodrilus hortensis* (Stephenson, 1910)	**−**	**−**	**−**	**−**	**−**	**−**	No voucher	Netherlands, Utrecht, Overvecht, city canal along Moldaudreef	52.1156 N	5.1261 E	4‐September‐2006	M. Vilhelm
CE12487	Naididae	*Branchiura* sp (undescribed)	**+**	**+/−**	>118	153	642	KY982553	No voucher	China, Hubei, Wuhan, Donghu Lake	30.55 N	114.358 E	15‐June‐2011	Hong‐zhu Wang
CE112	Naididae	*Clitellio arenarius* (Müller, 1776)	**+**	**+**	380	153	302	KY637031	No voucher	Sweden, Bohuslän, Strömstad, Tjärnö, Tjärnöviken, subtidal sand	58.876 N	11.145 E	1‐November‐1998	Christer Erséus
CE138	Naididae	*Doliodrilus tener* Erséus, 1984	**+**	**+**	>669	153	282	KY637032	No voucher	China, Hainan, E of Sanya City, fish pond at road to Teng Hai, brackish water, coarse sand with black mud	18.28 N	109.73 E	16‐March‐2000	Christer Erséus
CE14133	Naididae	*Doliodrilus tener* Erséus, 1984	**+**	**+**	>573	153	256	KY982557	SMNH 162136	Hong Kong, New territories, Mai Po marshes	22.49 N	114.03 E	1‐December‐2011	Qiu Jian‐wen
CE754	Naididae	*Epirodrilus pygmaeus* (Hrabě, 1935)	**+/−**	**−**	**−**	>70	>84	KY982558	SMNH 82594	Czech Republic, about 60 km W of Brno, Rokytnà village, Rokytnà River (Thay River basin)	49.17 N	15.79 E	1‐May‐2004	Jana Schenkova
CE236	Naididae	*Heronidrilus fastigatus* Erséus & Jamieson, 1981	**+**	**+**	444	153	285	KY637033	SMNH 160321	New Caledonia, Loyalty Islands, Lifou, Baie de Chataeubriand, Wé, 0.5 m, marine, medium sand;	20.55 S	167.17 E	21‐November‐2000	Christer Erséus
CE18212	Naididae	*Heronidrilus gravidus* Erséus, 1990	**+**	**+**	>472	153	247	KY982562	SMNH 162137	Belize, off Dangriga, sand bores area between Carrie Bow Cay and Wee Wee Cay, 2 m	16.7589 N	88.1127 W	13‐April‐2013	Judith Zimmermann, Cecilia Wentrup & Christer Erséus
CE17490	Naididae	*Heterodrilus ersei* (Giere, 1979)	**+**	**+**	>377	153	296	KY982563	SMNH 162138	Bahamas, Exuma, Norman's Pond Cay, lagoon outlet channel, coarse sand,	23.7681 N	76.1313 W	2‐April‐2013	Christer Erséus
CE18015	Naididae	*Inanidrilus leukodermatus* (Giere, 1979)	**+**	**+**	416	153	258	KY982564	SMNH 162139	Belize, off Dangriga, Carrie Bow Cay, seagrass bed, shallow subtidal, fine sand	16.8030 N	88.0812 W	11‐April‐2013	Judith Zimmermann, Cecilia Wentrup & Christer Erséus
CE131	Naididae	*Limnodriloides anxius* Erséus, 1990	**+**	**+**	>880	153	353	KY637034	No voucher	Bahamas, Exuma, Lee Stocking Island, subtidal sand	23.77 N	76.10 W	20‐April‐1999	Christer Erséus
CE16954	Naididae	*Limnodriloides australis* Erséus, 1982	**+**	**+**	>876	153	312	KY982566	SMNH 162140	Australia, Queensland, Heron Island	23.44528 S	151.91316 E	31‐August‐2012	Cecilia Wentrup, Manuel Kleiner & C. Erséus
CE2730	Naididae	*Limnodrilus cf. cervix* Brinkhurst, 1963	**+**	**+**	>480	153	408	KY982567	SMNH 162141	Sweden, Västergötland, Alingsås, Anten Lake, shallow water, sand	57.9911 N	12.4072 E	4‐August‐2007	Christer Erséus
CE2128	Naididae	*Limnodrilus claparedianus/cervix* (see Liu, et al., 2017)	**+**	**+**	378	153	346	KY369387	SMNH 159226	Germany, Osnabrück, lab culture at Zool Dep, Univ Osnabrück	52.283 N	8.033 E	16‐November‐2006	Annette Bergter
CE1785	Naididae	*Limnodrilus grandisetosus* Nomura, 1932	**+**	**+**	471	153	515	KY637016	SMNH 160311	Indonesia, Central Kalimantan, Tehang Lake	2.029 S	113.934 E	21‐March‐2005	Akifumi Ohtaka
CE1786	Naididae	*Limnodrilus grandisetosus* Nomura, 1932	**+**	**−**	−	>107	359	KY637017	SMNH 160312	Japan, Shimosakamoto, south basin of Biwa Lake	35.053 N	135.891 E	13‐February‐2003	Akifumi Ohtaka
CE1784	Naididae	*Limnodrilus hoffmeisteri* Claparède, 1862 (s.str., IX) (See Liu, et al., 2017)	**+**	**+**	341	153	341	KY369406	SMNH 159141	Japan, Akita‐ken, Minamiakita‐gun, Gojōme‐machi, Akita Prefecture, Lake Hachiro‐gata	39.933 N	140.082 E	9‐July‐2005	Akifumi Ohtaka
CE22814	Naididae	*Limnodrilus hoffmeisteri* II (See Liu, et al., 2017)	**+**	**−**	**−**	>70	342	KY652931	SMNH 158977	Switzerland, Chêne‐Bougeries, Chemin de la Montagne 22C, Seymaz River, organic (mostly leaf) matter (10‐25 cm)	46.199 N	6.194 E	24‐August‐2014	Yingkui Liu
CE2740	Naididae	*Limnodrilus hoffmeisteri* VIII (see Liu, et al., 2017)	**+**	**+**	349	153	328	KY369440	SMNH 159126	Sweden, Västergötland, Vårgårda, Lången Lake, 0.5‐1 m, sand	57.997 N	12.587 E	9‐August‐2007	Christer Erséus
CE1991	Naididae	*Limnodrilus hoffmeisteri* X (see Liu, et al., 2017)	**+**	**+**	338	153	340	KY369446	SMNH 159181	Sweden, Västergötland, Vårgårda, Lången Lake, shallow water	58.011 N	12.582 E	7‐August‐2006	Christer Erséus
CE10781	Naididae	*Limnodrilus rubripenis* Loden, 1977	**+**	**+**	547	153	422	KY637018	SMNH 160313	USA, Louisiana, Tangipahoa Co, Tangipahoa River at bridge on Road 10, near Arcola, sandy river bank	30.777 N	90.498 W	16‐January‐2011	Christer Erséus
CE10853	Naididae	*Limnodrilus rubripenis* Loden, 1977	**+**	**+**	550	153	432	KY637020	SMNH 160315	USA, Louisiana, Washington Co., Silver Creek, at bridge near Mount Hermon, muddy sand on banks and in water	30.971 N	90.289 W	17‐January‐2011	Christer Erséus
CE10482	Naididae	*Limnodrilus sulphurensis* Fend, Liu & Erséus, 2016	**+**	**+**	>589	153	383	KY637022	DMNS ZE.46275	USA, Colorado, Routt Co, City of Steamboat Springs, Sulfur Cave, high H2S stream in dark zone	40.48 N	106.75 W	11‐April‐2010	David Steinmann & Fred Luiszer
CE1839	Naididae	*Limnodrilus udekemianus* Claparède, 1862	**+**	**+**	>524	153	392	KY982568	SMNH 162142	Sweden, Småland, Jönköping, Strömsbergsbäcken Stream	57.753 N	14.182 E	21‐May‐2006	Daniel Gustafsson
CE211	Naididae	*Lophochaeta ignota* Štolc, 1886	**+**	**+/−**	>188	153	439	KY637036	No voucher	Sweden, Västergötland, Vårgårda, Lången Lake	57.997 N	12.887 E	1‐October‐2000	Christer Erséus
CE20081	Naididae	*Monopylephorus irroratus* (Verrill, 1873)	**+**	**+**	>370	153	330	KY982571	No voucher	Norway, Östfold, Fredrikstad, Öyenkilen, marina at Öyenkilveien, seashore, brackish(?)	59.1733 N	10.8485 E	23‐September‐2013	Christer Erséus
CE50	Naididae	*Monopylephorus rubroniveus* Levinsen, 1884	**+**	**+**	>435	153	370	KY637037	No voucher	Sweden, Södermanland, Nynäshamn, Torö, seashore	58.84 N	17.87 E	1‐September‐1998	Michael Norén
CE19318	Naididae	*Nais elinguis* Müller, 1774	**+**	**+**	>421	153	292	KY982572	No voucher	Norway, Sogn og Fjordane, Luster, Nes, seashore	61.3864 N	7.3691 E	12‐August‐2013	Christer Erséus
CE16885	Naididae	*Olavius albidus* (Jamieson, 1977)	**+**	**+**	>400	153	274	KY982573	SMNH 162143	Australia, Queensland, Heron Island	23.4434 S	151.9131 E	30‐August‐2012	Cecilia Wentrup, Manuel Kleiner & C. Erséus
CE17410	Naididae	*Potamothrix bavaricus* (Oschmann, 1913)	**+**	**+**	>479	153	383	KY982574	No voucher	Australia, Western Australia, S of Dunsborough, about 20 km S of Yallingup, near Woodlands, Wilyabrup Brook at Caves Road, stream	33.7948 S	115.0313 E	17‐September‐2012	Christer Erséus, Adrian Pinder & Yongde Cui
CE283	Naididae	*Potamothrix moldaviensis* Vejdovský & Mrázek, 1903	**+**	**+**	>466	153	396	KY637042	No voucher	Estonia, Rannu, Vörtsjärv Limnological Station, lab culture kept by Tarmo Timm	58.212 N	26.110 E	1‐December‐2000	Timm Tarmo
CE2883	Naididae	*Psammoryctides albicola* (Michaelsen, 1901)	**+**	**+**	>487	153	518	KY637043	SMNH 160323	Sweden, Södermanland, Österåker, Vingåker, Låttern Lake, sand near shore	59.0854 N	16.0426 E	30‐July‐2007	Christer Erséus
CE289	Naididae	*Psammoryctides barbatus* (Grube, 1861)	**+**	**+**	391	153	373	KY637044	No voucher	Estonia, Rannu, Vörtsjärv Limnological Station, lab culture kept by Tarmo Timm	58.212 N	26.110 E	1‐December‐2000	Timm Tarmo
CE623	Naididae	*Rhyacodrilus coccineus* (Vejdovský, 1875)	**+**	**+**	340	153	308	KF267996	No voucher	Sweden, Västergötland, Vårgårda, stream between Iglasjön and Lången Lakes, sand	58.0103 N	012.5836 E	6‐July‐2003	Christer Erséus
CE17550	Naididae	*Smithsonidrilus hummelincki* (Righi & Kanner, 1979)	**+**	**+/−**	>69	153	697	KY982576	SMNH 162144	Bahamas, Exuma, Little Darby Island, in front of Research Station, intertidal sand	23.8558 N	76.2248 W	4‐April‐2013	Christer Erséus
CE1984	Naididae	*Spirosperma ferox* Eisen, 1879	**+**	**−**	**−**	>69	>306	KY982577	SMNH 162145	Sweden, Västergötland, Vårgårda, Lången Lake, shallow water	58.011 N	12.582 E	6‐August‐2006	Christer Erséus
CE18140	Naididae	*Thalassodrilides bruneti* Erséus, 1990	**+**	**+**	>514	153	281	KY982579	SMNH 153613	Belize, off Dangriga, Carrie Bow Cay, shallow subtidal, 0.7 m	16.803 N	88.082 W	12‐April‐2013	Judith Zimmermann, Cecilia Wentrup & Christer Erséus
CE2038	Naididae	*Trieminentia corderoi* (Harman, 1970)	**+/−**	**−**	**−**	>63	>161	KY982580	SMNH 104788	Argentina, Entre Ríos, NW of Paraná City, floodplain lake connected to Middle Paraná River	31.665 S	60.590 W	18‐August‐2006	Mercedes Marchese
CE2044	Naididae	*Tubifex blanchardi* Vejdovský, 1891	**+**	**+**	>604	153	446	KY637046	SMNH 160324	Belgium, Oost‐Vlaanderen, near Schoonaarde, Paddebeek River	51.02 N	4.05 E	7‐September‐2006	Jan Soors
CE272	Naididae	*Tubifex newaensis* (Michaelsen, 1903)	**+**	**+**	>490	153	336	KY637047	No voucher	Estonia, Rannu, Vörtsjärv Limnological Station, lab culture kept by Tarmo Timm	58.212 N	26.110 E	1‐December‐2000	Timm Tarmo
CE212	Naididae	*Tubifex smirnowi* Lastockin, 1927	**+**	**+**	447	153	321	KY637048	No voucher	Sweden, Västergötland, Vårgårda, Lången Lake	57.997 N	12.887 E	13‐July‐2002	Christer Erséus
CE276	Naididae	*Tubifex tubifex* (Müller, 1774)	**+**	**+**	>515	153	393	KY637049	No voucher	Originally from Kyrgyzstan Republic, Frunze (Bisjkek); kept in Timm's lab culture	42.85 N	74.37 E	1‐December‐2000	Timm Tarmo
CE186	Naididae	*Tubificoides benedii* (Udekem, 1855)	**+**	**+**	>464	153	332	KY637050	No voucher	Sweden, Bohuslän, Strömstad, Tjärnö, at Research Station, intertidal sand	58.876 N	11.146 E	1‐September‐2000	Christer Erséus
CE3600	Naididae	*Varichaetadrilus cf. angustipenis* (Brinkhurst and Cook, 1966)	**−**	**−**	**−**	**−**	**−**	**−**	SMNH 160325	USA, Alabama, Madison County, Huntsville, WEUP Radio Station Pond	34.7603 N	86.6431 W	17‐March‐2008	Christer Erséus & Mark Wetzel
CE3621	Naididae	*Varichaetadrilus* sp (see Liu, et al., 2017)	**+**	**+/−**	**−**	>105	494	KY637051	SMNH 160326	USA, Alabama, Madison County, Huntsville, WEUP Radio Station Pond	34.7603 N	86.6431 W	17‐March‐2008	Christer Erséus & Mark Wetzel
CE14357	Phreodrilidae	*Antarctodrilus proboscidea* (Brinkhurst & Fulton, 1979)	**+**	**−**	**−**	>85	747	KY982548	No voucher	Australia, Victoria, Acheron River (NE of Melbourne), gravel and sand	37.3526 S	145.7066 E	12‐April‐2012	C. Erséus & Richard Marchant
CE14476	Randiellidae	*Randiella* sp (undescribed)	**+**	**+**	>548	153	319	KY982575	No voucher	Australia, Queensland, Lizard Island, Watson's Bay, Ferrier's Creek, brackish water	14.666 S	145.451 E	20‐April‐2012	Christer Erséus

The entire ITS and the ITS2 sequences were amplified, each with its new primer pair. The PCR reaction mixtures consisted of 15 μl of VWR red Taq Master Mix kit (We Enable Science, Denmark), 1 μl of primer (10 mmol/l), 2 μl of DNA template, and 6 μl distilled water. The PCR protocol for both pairs was as follows: initial denaturation at 95°C for 5 min; 35 cycles of denaturation at 95°C for 45 s, annealing at 55°C for 60 s and elongation at 72°C for 90 s, followed by a final extension at 72°C for 8 min. Gel electrophoresis (1% agarose in 10 × TAE buffer) was carried out to check the quality of PCR products, which were then were purified using 5 μl ExoTAP (Exonuclease I and FastAP Thermosensitive Alkaline Phosphatase). Amplicons were sequenced by Eurofins (Germany). For both of the new primer pairs, amplicons at least 200 bp long were regarded as successful. The amplified sequences were then checked for adherence to clitellates by blasting them against the NCBI database.

### 2.3 Primer evaluation in silico

The specificity of the new primers to clitellates (relative to other organisms) was evaluated in silico by the number of mismatches between DNA templates and primers, and the results of this were also compared with the specificity of primers previously used in clitellate studies (Figure 2). These analyses were performed using ecoPCR (Ficetola et al., 2010) against assembled and annotated sequences (STD, version r127) in EMBL. To achieve simulation under realistic PCR conditions, up to three mismatches between a primer and its annealing sequence were allowed. The complete length of clitellate ITS sequences at NCBI normally varies between 500 and 900 bp; however, members of Branchiobdellida have a rather long (about 1200 bp) ITS1 spacer (Williams, Gelder, Proctor, & Coltman, 2013). Thus, in the simulations, sizes of ITS (as a whole) between 400 and 2500 bp were allowed, and the minimum and maximum amplified ITS2 lengths were set as 200 and 1250 bp long, respectively.

## 3 RESULTS

### 3.1 Annotation of ITS sequences and primer design

As mentioned above, 742 GenBank sequences, representing a total of at least 46 genera belonging to 14 clitellate families (Table S1), were obtained, annotated, and aligned. As expected, in this alignment, sequence variation is much greater in the ITS spacers than in the 18S, 5.8S, and 28S rDNA partitions. The majority of the published complete 5.8S sequences contain 153 ± 1 nucleotides. Figure 1 shows the variations in a part of 5.8S among the 27 haplotypes found in our newly amplified complete ITS sequences, with taxa ranked by number of mismatches. Neither the first nor the third of the three conserved 5.8S motifs proposed by Harpke and Peterson (2008) are identical with our current clitellate ones (Figure 2: CM1 and CM3), but in most cases the second motif (Figure 2: CM2) is the same as the conserved motif in vertebrates (Harpke & Peterson, 2008). The complete ITS2 spacer, recognized by the 5.8S–28S rDNA interaction (Keller et al., 2009), varied from 174 to 503 bp in the current clitellate sample. The motif CATTA was identified as the end of 18S by the software ITSx, and this ending motif was found in eukaryote sequences from the Rfam database. In addition, it also has been found that, in some fungi, the ITS1 spacer starts after this motif CATTA (Nagy et al., 2012; Schoch et al., 2014). The complete ITS1 sequences, which begin after the conserved motif CATTA, ranged from 314 to 1117 bp in the published clitellate sequences.

Two new primer pairs suggested by Oligo 7, and now referred to as 29F/1084R and 606F/1082R, were found to be suitable for amplifications of the whole ITS region, and the ITS2 subregion, respectively, of Clitellata. The forward primer 29F (AAAGTCGTAACAAGGTTTCCGTA) matches the terminal end of 18S but after E18S‐2, with its anchoring sites partly overlapping with those of the old primers ITS5 and ETTS2, and the reverse primer 1084R (YGTTAGTTTCTTTTCCTCCGCTT) partly overlaps with ITS4 but is separated from ETTS1 and E28S‐2 (Figure 2 and Figure S1). The new forward primer for ITS2, 606F (GTCGATGAAGAGCGCAGCCA), partly overlaps with ITS3 and 5.8SF but was designed to fully match the motif CM1 (Figure 1), and the corresponding reverse primer, 1082R (TTAGTTTCTTTTCCTCCGCTT), is almost identical to 1084R (Figure 2 and Figure S1), but two nucleotides shorter at the 5′ end, which makes its melting properties similar to those of 606F.

### 3.2 Experimental verification of new primers

From our 71 genomic samples, 52 (73%) ITS amplicons were successfully amplified using the primer pair 29F/1084R, and 65 (91.5%) ITS2 amplicons were successfully amplified using 606F/1082R. Sequences are deposited in the NCBI database (for more details see Table 2). All samples that gave no amplifications, and those that yielded amplicons <200 bp long, also failed in PCR reactions using only one universal primer pair ITS5/ITS4. Successfully amplified ITS and ITS2 sequences from the same individual were identical in their overlapping parts, after trimming. The average GC content of the successfully amplified ITS and ITS2 sequences was around 59%, but amplicon lengths varied significantly across taxa. After trimming, the completely amplified ITS sequences using 29F/1084R spanned from 844 to 1439. But in one case, KY982581 (a branchiobdellidan *Xironogiton victoriensis* CE18252), the length was 2,060 bp, and yet this ITS region was not completely amplified. ITS2 amplicons using 606F/1082R ranged from 329 to 912 bp, and they often include parts of 5.8S and 28S sequences. The complete 5.8S for CE1790 (a naidid, *Aulodrilus acutus*, KY637027) was 154 bp, while all other complete sequences of 5.8S were 153 bp; all new 5.8S are consistent with the published 5.8S sequences in length. The completely amplified ITS1 spacer ranged from 351 to 733 bp, whereas the ITS2 spacer varied from 247 bp to 747 bp. The amplified ITS1, even incomplete ones, was generally longer than ITS2 of the same individual or a closely related species (see Table 2).

Interestingly, our attempt to amplify ITS of *Chamaedrilus sphagnetorum* (CE11317) using the new primer pair 29F/1084R failed, while a 909‐bp‐long ITS sequence (KF672519) was successfully amplified from the same individual using two pairs of primers (Martinsson & Erséus, 2014). Nevertheless, our new ITS2 amplicon (primers 606F/1082R) of this worm is identical to the corresponding part in KF672519.

The mismatches between the primers and their targeting 5.8S were investigated (see Figure 2 and Figure S1). The primers 5.8SF, 5.8SR, ITS3, and ITS1B often had more than one mismatch against the amplified DNA sequences, while 606F showed only one mismatch with the sequences from Haplotaxidae (CE5731, *Haplotaxis gordioides*) and Haemopidae (CE18378, *Haemopis sanguisuga*). For all other sequences of our samples of clitellates, 606F showed a 100% match with its annealing region.

### 3.3 Primer evaluation in silico

**Figure 3:**
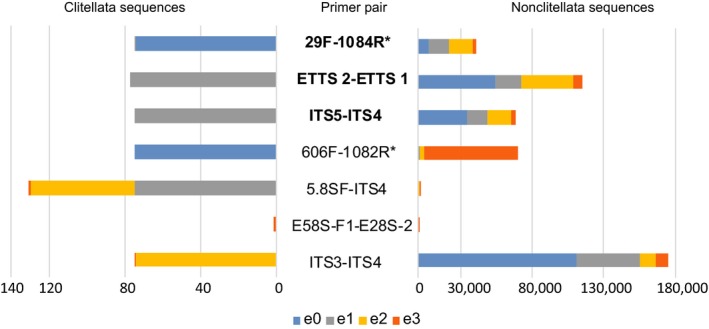
In silico PCR output, that is, numbers of GenBank ITS and ITS2 sequences amplified, using different primers pairs, and allowing 0–3 nucleotide mismatches between published sequences and primers. Primers for amplifying complete ITS sequences are in bold face, those for ITS2 are not, and the newly designed pairs are marked with an asterisk (*). The colors blue, gray, yellow, and orange, respectively, separate sequences with no mismatches with primers (e0), or, for at least one primer in the pair, with 1 mismatch (e1), 2 mismatches (e2), or 3 mismatches (e3). The cumulative bars on the left side show numbers of in silico amplified clitellate sequences only, the ones on the right side show the sequences of all other (i.e., nonclitellate) organisms

The in silico results varied considerably across simulations with different primer pairs (Figure 3 and Table S2). Generally, only a few ITS sequences of clitellates were successfully (in silico) amplified due to the limited number of full‐length ITS sequences available. A much larger number of nonclitellate amplicons come from fungal groups, in particular, followed by, for example, chlorophytes (green algae) and some of the more species‐rich invertebrate groups, such as Cnidaria, Nematoda, Arthropoda, and Plathyhelminthes (Table S2). Under strict PCR conditions (0–1 mismatch for each primer), about 70 clitellate sequences of the complete ITS region were amplified in silico with ETTS2/ETTS1, ITS5/ITS4, and the new primer pair 29F/1084R (Figure 3). On the other hand, even under more relaxed PCR conditions (up to three mismatches per primer), the number of nonclitellate amplicons was dramatically decreased when using 29F/1084R instead of ETTS2/ETTS1 and ITS5/ITS4. For the evaluation of ITS2 primers and their specificity for clitellates, 606F/1082R and 5.8FS/ITS4 did better than ITS3/ITS4 and E58S‐F1/E28S‐2 under the strict conditions (0–1 mismatch). Under relaxed PCR conditions (2–3 mismatches), a higher number (131) of clitellate ITS2 sequences were amplified with 5.8FS/ITS4, and a similar number of ITS2 amplicons for the primer pairs 606F/1082R and ITS3/ITS4. The amplified nonclitellate sequences using 5.8FS/ITS4 were also fewer than those using 606F/1082R, and even fewer than those using ITS3/ITS4.

In addition, the possible mismatches between each primer and the haplotypes of the corresponding template regions in the newly amplified (Figure 2) and previously published clitellate ITS sequences were estimated, and differences in all these mismatches (number and position) are summarized in Figure S1.

## 4 DISCUSSION

### 4.1 Annotation of ITS

When using ITS for phylogenetic analysis, verification and annotation of amplicons are critical. Nonfunctional pseudogenes or chimeric sequences are readily recognizable by irregularities in the 5.8S rDNA and/or by the absence of some or all of the conserved regions of the ITS spacers (Freire et al., 2012; Harpke & Peterson, 2008; Hřibová et al., 2011; Rampersad, 2014). Only the GenBank clitellate sequences with recognizable 5.8S region were selected for primer design. Many such published ITS sequences are commonly co‐amplified with some rDNA residues, but the various parts of the (18S)‐ITS1‐5.8S‐ITS2‐(28S) sequences are neither properly annotated nor partitioned. It is widely accepted that an accurate alignment of positional homologies is highly important for the final phylogenetic reconstruction (Katoh & Standley, 2013; Ogden & Rosenberg, 2006). However, indel events make multiple alignment of divergent ITS sequences challenging, due to a high risk of inferring false‐positive positional homologies and increasing artefactual support for incorrect relationships (Nagy et al., 2012). In particular, when incomplete ITS sequences are included in an alignment, short unannotated 18S and 28S residues are prone to misalign with highly variable ITS spacer sequences. Moreover, if residues are <25 nucleotides long, annotation of ITS sequences with short adjacent residues of 18S and 28S rDNA is problematic (Bengtsson‐Palme et al., 2013; Nagy et al., 2012). Our new ITS sequences, amplified from 11 clitellate families, are meant to be used as references to improve annotation of similar amplicons in the future.

### 4.2 Limitations of universal ITS primers

Universal ITS primers do not perfectly match their annealing template sequences of all organisms (see https://unite.ut.ee/primers.php). Even for the well‐studied Kingdom Fungi, it is difficult to amplify the whole ITS region of all groups using a single universal primer pair (Konieczny, Roterman‐Konieczna, & Spólnik, 2014). The in silico analyses of published data showed that the ITS primers traditionally used for clitellates are neither universal nor efficient enough for this group; for example, the primer 5.8SF may have up to five mismatches with its template DNA (Figure 2). Although this result may have been biased by the limited number of clitellate sequences (and lacking representation of some families) in the EMBL database, we also observed notable mismatches (Figure S1) between the newly amplified complete ITS sequences (using 29F/1084R) and primers targeting 5.8S rDNA: E58S‐F1, ITS3, 5.8SF, 5.8SR, ITS1B, and E58S‐R1 (see also Figure 2). Unfortunately, there is not much information about the flanking 18S rDNA (Figure S1) to optimize the specific clitellate primers for amplification of the whole ITS region. Still, however, as noted above, Martinsson and Erséus (2014) obtained a 909‐bp ITS sequence (KF672519) from the DNA extract of an enchytraeid (CE11317) using the universal primer pair ITS5/ITS4, but for which we failed when using 29F/1084R. This may be explained by the former authors’ use also of 5.8SF/5.8SR, which in this case only show a few mismatches with KF672519.

For primers, in general, even one or a few mismatches between primer and DNA template may jeopardize amplification (Bellemain et al., 2010; Bru, Martin‐Laurent, & Philippot, 2008; Huang, Arnheim, & Goodman, 1992; Ihrmark et al., 2012; Wright et al., 2014; Wu, Hong, & Liu, 2009). In addition, especially for clitellates feeding on plant material and fungi (Bonkowski, Griffiths, & Ritz, 2000; Curry & Schmidt, 2006; Uchida et al., 2004), it could be hypothesized that universal primers may amplify fragments of contaminating plant or fungal sequences instead of sequences of clitellates. However, it is likely to avoid, or at least minimize, contamination, and also amplification of pseudogene sequences, using the new primer 606F, which targets a specific conservative motif in the clitellate 5.8S.

The sensitivity of PCR success rate to primer mismatches probably needs further investigation, but amplification of GC‐rich ITS sequences may be improved by following a combination strategy of adding enhancers and modifying the PCR cycle conditions (Mamedov et al., 2008; Sahdev, Saini, Tiwari, Saxena, & Singh Saini, 2007). In our case, however, the GC contents of the whole ITS and its partial ITS2 sequence are almost equal. It seems that the length of target loci is more critical for successful amplification and sequencing than any of the other factors mentioned above. To use a single primer pair to amplify ITS sequences longer than about 1,500 bp is challenging. Thus, to choose one of the generally much shorter ITS spacers (with flanking rDNAs providing reliable primer templates) may be the optimal option for broad samples of clitellate taxa.

### 4.3 Choosing primers

Although only two‐thirds of the clitellate samples were successfully amplified using the primer pair 29F/1084R, the in silico test showed that the specificity of this primer pair is better than that of ITS5/ITS4 and ETTS1/ETTS2 (Figure 3). Therefore, when this pair proves to work for some clitellate taxa, it is likely to be a good option for sequencing the ITS region as a whole; that is, if it is <about 1,500 bp long.

The in silico results not only give a hint about the relative performance of commonly used and new ITS primer pairs, but they also predict potential nontarget amplicons and length of amplicons before selecting a primer pair for studies of a specific clitellate group. In the in silico test of different ITS2 primers, 5.8SF/ITS4 theoretically performed better than 606F/1082R, that is, the former pair amplified more clitellate sequences and less nonclitellate sequences than the latter (Figure 3). However, this was only under rather relaxed conditions (2–3 mismatches allowed). Moreover, poor specificity of the 5.8SF (as shown in the Figure S1), originally designed for bivalves (Källersjö et al., 2005), limits the potential number of ITS2 amplicons. Because of this, while ITS5/ITS4 produced almost 70,000 nonclitellate ITS amplicons, 5.8SF/ITS4 could only generate a very low number of ITS2 amplicons (Figure 3). On the other hand, 606F, targeting a conservative and unique 5.8S motif of clitellates, was much more specific than any of the older primers for clitellates (Figure 2; Figure S1). The pair 606F/1082R also had a low success rate in silico amplifications of nonclitellate groups (Figure 3). Therefore, this new primer pair is more suitable than other published primers to amplify the ITS2 regions from a taxonomically broad range of clitellates.

The primer with a 3′‐terminal “A” nucleotide, that is, our new primers 29F, 606F, and 1082R, may be less efficient in amplifications using Taq DNA polymerase, regardless of the corresponding nucleotide in the template strand (Arezi, Xing, Sorge, & Hogrefe, 2003; Ayyadevara, Thaden, & Shmookler Reis, 2000). Therefore, alternative polymerases may help to increase the success rate for some clitellate specimens. For some polyploid clitellates (e.g., within Lumbricidae, Enchytraeidae, and Naididae (see Casellato, 1984; Gregory & Hebert, 2002) with multiple copies of the ITS region, however, sequencing using our new primer may still be challenging. This is because the Sanger sequencing method can only be performed on a single pure amplicon. Using a particular PCR primer pair to amplify multiple copies of a gene may lead to double peaks in the chromatograms at sites that differ between the copies. The PCR may even fail completely because all sites after indels (introns leading to sequence length differences) will produce seemingly undecipherable double peaks (Griffin, Robin, & Hoffmann, 2011). In such cases, the software Champuru (http://seqphase.mpg.de/champuru/), which is able to detect and separate the gene copies, may be useful for diploids (Flot, 2007), while cloning or Next Generation Sequencing may be more practical tools for polyploids (Aversano et al., 2012; Brassac & Blattner, 2015; Griffin et al., 2011).

## 5 CONCLUSION

This study has shown that the new primer pair 606F/1082R has great specificity in amplification of the ITS2 of Clitellata, at least for the 18 families investigated by either in vitro or in silico analyses: Bdellodrilidae, Branchiobdellidae, Cambarincolidae, Capilloventridae, Enchytraeidae, Erpobdellidae, Glossiphoniidae, Glossoscolecidae, Haemadipsidae, Haemopidae, Haplotaxidae, Hirudinidae, Lumbricidae, Lumbriculidae, Megascolecidae, Naididae, Phreodrilidae, and Randiellidae. This will facilitate many kinds of molecular systematic studies of this common and ecologically important group of worms. The other pair, 29F/1084R amplifying the whole ITS, will be a useful complement to existing ITS primers.

## CONFLICT OF INTEREST

None declared.
