# Supplementary material for: New specific primers for amplification of the Internal Transcribed Spacer region in Clitellata (Annelida)
Source: Ecol Evol. 2017 Oct 31;7(23):10421–39. doi: 10.1002/ece3.3212 (PMC5723599; doi:10.1002/ece3.3212)
Supplement: Supplementary file 1 [file ECE3-7-10421-s001.pdf]

Supplementary Fig. S1 Primers compared with the haplotypes of the corresponding template regions

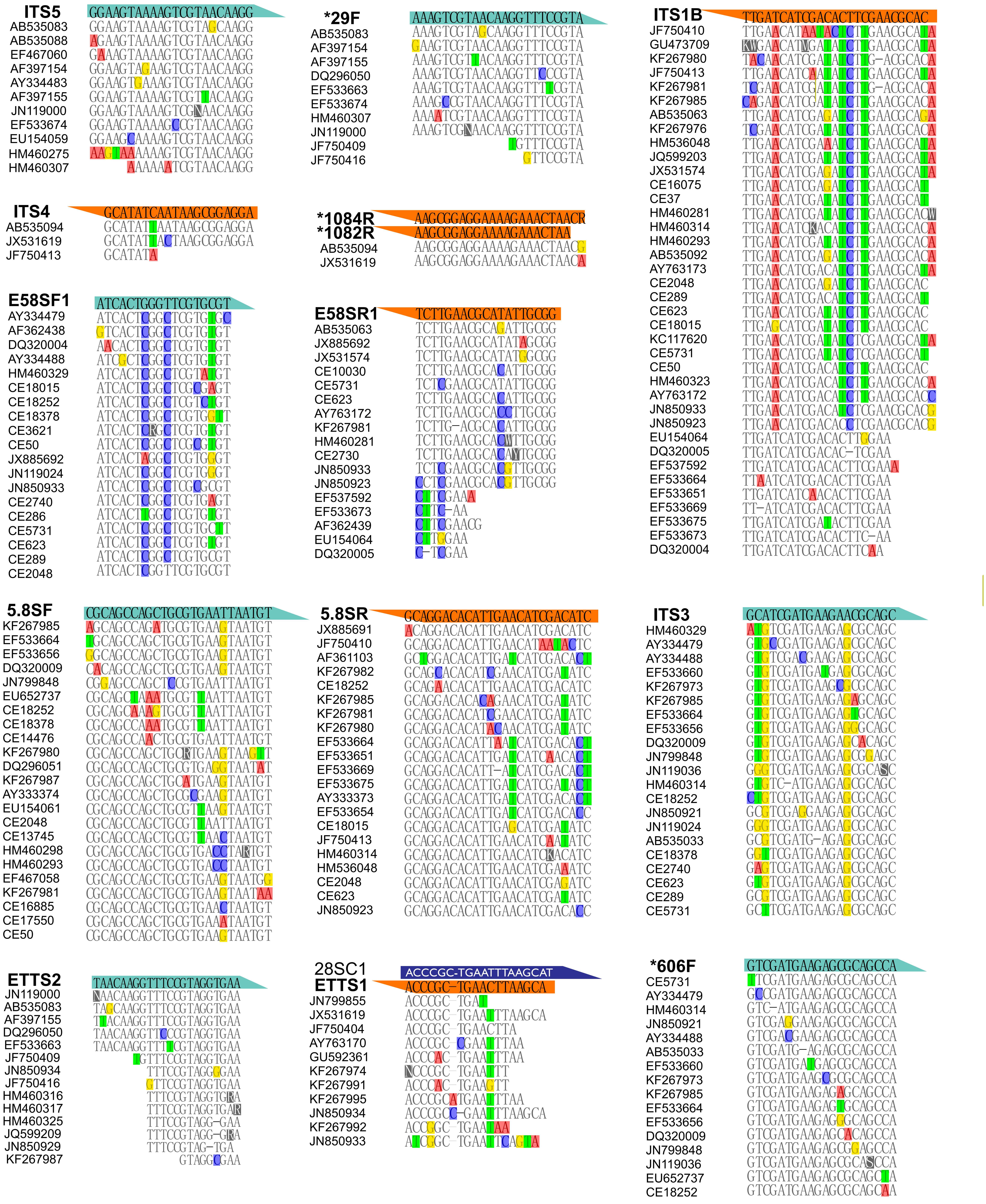

Mismatches between primers and known DNA templates are highlighted (the substitution T in green, A in red, C in blue and G in yellow). The new amplified sequences start with "CE", and remaining are Genbank access numbers for the published sequences. The forward (cyan arrows) and reverse primers (orange arrows) of newly designed (start with an asterisk) and previously published primers (without an asterisk) were marked. In addition, the commonly used primer 28SC1 (Jamieson et al. 2002) (purple arrow) for amplifying 28S, the reverse of ETTS1, is also show here.
