# Supplementary material for: New specific primers for amplification of the Internal Transcribed Spacer region in Clitellata (Annelida)
Source: Ecol Evol. 2017 Oct 31;7(23):10421–39. doi: 10.1002/ece3.3212 (PMC5723599; doi:10.1002/ece3.3212)
Supplement: Supplementary file 2 [file ECE3-7-10421-s002.pdf]

Supplementary Table S1.

Taxonomy summary of the published ITS clitellate sequences in the NCBI database

Family	Genus	Number
Bdellodrilidae	*Cronodrilus*	1
Bdellodrilidae	*Uglukodrilus*	1
Branchiobdellidae	*Bdellodrilus*	1
Branchiobdellidae	*Magmatodrilus*	1
Branchiobdellidae	*Oedipodrilus*	1
Branchiobdellidae	*Pterodrilus*	7
Branchiobdellidae	*Sathodrilus*	6
Branchiobdellidae	*Triannulata*	1
Branchiobdellidae	*Xironogiton*	4
Cambarincolidae	*Cambarincola*	22
Enchytraeidae	*Bryodrilus*	5
Enchytraeidae	*Fridericia*	1
Enchytraeidae	*Grania*	49
Enchytraeidae	*Lumbricillus*	6
Enchytraeidae	*Marionina*	21
Enchytraeidae	*Mesenchytraeus*	8
Erpobdellidae	*Erpobdella*	1
Glossiphoniidae	*Haementeria*	11
Glossiphoniidae	*Placobdella*	1
Glossoscolecidae	*Rhinodrilus*	23
Haemadipsidae	*Haemadipsa*	1
Haemopidae	*Haemopis*	1
Haemopidae	*Whitmania*	13
Hirudinidae	*Hirudo*	60
Hirudinidae	*Poecilobdella*	3
Lumbricidae	*Aporrectodea*	2
Lumbricidae	*Dendrodrilus*	1
Lumbricidae	*Eisenia*	41
Lumbricidae	*Lumbricus*	2
Lumbricidae	*Octolasion*	1
Lumbricidae	*unclassified*	1
Megascolecidae	*Amynthas*	1
Megascolecidae	*Duplodicodrilus*	1
Megascolecidae	*Metaphire*	73
Tubificidae (=Naididae)	*Ilyodrilus*	1
Tubificidae (=Naididae)	*Limnodrilus*	2
Tubificidae (=Naididae)	*Nais*	81
Tubificidae (=Naididae)	*Rhyacodrilus*	24
Tubificidae (=Naididae)	*Tubifex*	127
Tubificidae (=Naididae)	*Tubificoides*	60
Lumbriculidae	*Eclipidrilus*	3
Lumbriculidae	*Eremidrilus*	2
Lumbriculidae	*Guestphalinus*	1
Lumbriculidae	*Kincaidiana*	1
Lumbriculidae	*Lumbriculus*	18
Lumbriculidae	*Rhynchelmis*	27
Lumbriculidae	*Stylodrilus*	23
