# Supplementary material for: New specific primers for amplification of the Internal Transcribed Spacer region in Clitellata (Annelida)
Source: Ecol Evol. 2017 Oct 31;7(23):10421–39. doi: 10.1002/ece3.3212 (PMC5723599; doi:10.1002/ece3.3212)
Supplement: Supplementary file 3 [file ECE3-7-10421-s003.pdf]

Supplementary Table S2. Numbers of *in silico* amplified non-clitellate amplicons using different primer pairs

**ITS:** 29F/1084*	NOA	**ITS:** ETTS2/ETTS1	NOA	**ITS:** ITS5/ITS4	NOA	**ITS:** E18S-2/E28S-1	NOA	**ITS2:** 606F/1082R*	NOA	**ITS2:** 5.8SF/ITS4	NOA	**ITS2:** E58S-f1/E28s	NOA	**ITS2:** ITS3/ITS4	NOA	**ITS2:** byk4/ITS4	NOA	**ITS2:** skra4/ITS4	NOA
**In total**	37480	**In total**	94554	**In total**	57329	**In total**	32	**In total**	55767	**In total**	1923	**In total**	1288	**In total**	142582	**In total**	32	**In total**	9
Ascomycota	15159	Ascomycota	35407	Ascomycota	23101	Mollusca	32	Ascomycota	33082	Platyhelminthes	921	Platyhelminthes	465	Ascomycota	68157	Streptophyta	19	Chlorophyta	1
Streptophyta	8586	Streptophyta	24225	Streptophyta	14860	Streptophyta	1	Basidiomycota	14641	Mollusca	589	Nematoda	205	Streptophyta	29672	Chordata	4	Nematoda	8
Basidiomycota	4843	Basidiomycota	18075	Basidiomycota	9358			Nematoda	1746	Chordata	229	Chordata	198	Basidiomycota	29249	Ascomycota	4		
Glomeromycota	4231	Glomeromycota	7425	Glomeromycota	5605			Glomeromycota	1712	Nematoda	67	Arthropoda	113	Glomeromycota	5871	Arthropoda	2		
Nematoda	1278	Nematoda	2527	Cnidaria	1000			Cnidaria	1111	Annelida	34	Mollusca	100	Nematoda	2369	Basidiomycota	2		
Cnidaria	860	Cnidaria	1446	Arthropoda	683			Arthropoda	836	Echinodermata	29	Nematomorpha	54	Chlorophyta	1399	Apicomplexa	1		
Arthropoda	505	Arthropoda	1197	Chlorophyta	565			Platyhelminthes	642	Nemertea	21	Annelida	32	Cnidaria	1246				
Chlorophyta	486	Chlorophyta	1189	Platyhelminthes	458			Chordata	274	Bryozoa	19	Cnidaria	30	Arthropoda	1194				
Platyhelminthes	381	Platyhelminthes	637	Mollusca	315			Porifera	210	Cnidaria	7	Echinodermata	27	Platyhelminthes	718				
Apicomplexa	259	Apicomplexa	497	Apicomplexa	295			Mollusca	188	Hemichordata	4	Nemertea	21	Mollusca	571				
Phaeophyceae	204	Porifera	418	Porifera	218			Chlorophyta	131	Ascomycota	1	Bryozoa	19	Bacillariophyta	388				
Porifera	201	Mollusca	333	Phaeophyceae	208			Annelida	33	Brachiopoda	1	Actinobacteria	7	Apicomplexa	373				
Chordata	189	Chordata	282	Chordata	199			Echinodermata	29	Chaetognatha	1	Basidiomycota	7	Chordata	294				
Bacillariophyta	83	Phaeophyceae	234	Nematoda	145			Entorrhizomycota	25			Streptophyta	3	Phaeophyceae	275				
Nematomorpha	52	Bacillariophyta	233	Bacillariophyta	104			Nemertea	21			Hemichordata	2	Porifera	272				
Mollusca	36	Acanthocephala	65	Nematomorpha	52			Bryozoa	19			Proteobacteria	2	Neocallimastigomycota	236				
Annelida	31	Chytridiomycota	63	Annelida	30			Apicomplexa	15			Ascomycota	1	Nematomorpha	54				
Entorrhizomycota	21	Nematomorpha	52	Chytridiomycota	20			Blastocladiomycota	3			Brachiopoda	1	Chytridiomycota	36				
Bryozoa	19	Annelida	41	Entorrhizomycota	20			Ctenophora	3			Porifera	1	Annelida	33				
Eustigmatophyceae	10	Proteobacteria	33	Bryozoa	19			Hemichordata	2					Echinodermata	29				
Echinodermata	9	Entorrhizomycota	23	Neocallimastigomycota	19			Brachiopoda	1					Entorrhizomycota	26				
Bacteroidetes	8	Neocallimastigomycot	21	Entomophthoromycota	12			Chaetognatha	1					Nemertea	21				
Chytridiomycota	6	Bryozoa	19	Echinodermata	9			Placozoa	1					Bryozoa	19				
Colponemidia	3	Verrucomicrobia	17	Blastocladiomycota	8									Entomophthoromycota	16				
Blastocladiomycota	2	Blastocladiomycota	16	Eustigmatophyceae	6									Blastocladiomycota	14				
Crenarchaeota	2	Entomophthoromycot	12	Picozoa	4									Eustigmatophyceae	11				
Ctenophora	2	Euryarchaeota	10	Colponemidia	3									Placozoa	10				
Firmicutes	2	Eustigmatophyceae	10	Proteobacteria	3									Proteobacteria	7				
Proteobacteria	2	Echinodermata	9	Ctenophora	2									Picozoa	6				
Verrucomicrobia	2	Chromerida	8	Hemichordata	2									Hemichordata	4				
Chlamydiae	1	Firmicutes	6	Chromerida	1									Colponemidia	3				
Chromerida	1	Picozoa	6	Cryptomycota	1									Ctenophora	3				
Cryptomycota	1	Rotifera	4	Haplosporidia	1									Brachiopoda	1				
Entomophthoromycota	1	Colponemidia	3	Microsporidia	1									Chaetognatha	1				
Fusobacteria	1	Ctenophora	3	Rotifera	1									Chromerida	1				
Microsporidia	1	Aquificae	2	Xanthophyceae	1									Cryptomycota	1				
Neocallimastigomycota	1	Hemichordata	2											Pinguiophyceae	1				
Picozoa	1	Cryptomycota	1											Xanthophyceae	1				
		Haplosporidia	1																
		Nitrospirae	1																
		Xanthophyceae	1																

NOA is the number of amplicons, the new primer pairs are marked with an asterisk (*).
